# Contracting eastern African C_4_ grasslands during the extinction of *Paranthropus boisei*

**DOI:** 10.1038/s41598-021-86642-z

**Published:** 2021-03-30

**Authors:** Rhonda L. Quinn, Christopher J. Lepre

**Affiliations:** 1 Department of Sociology, Anthropology, Social Work and Criminal Justice, Seton Hall University, 400 South Orange Ave, South Orange, NJ 07079 USA; 2 Department of Earth and Planetary Sciences, Rutgers University, 610 Taylor Road, Piscataway, NJ 08854 USA

**Keywords:** Anthropology, Palaeoclimate, Evolution, Palaeoecology

## Abstract

The extinction of the *Paranthropus boisei* estimated to just before 1 Ma occurred when C_4_ grasslands dominated landscapes of the Eastern African Rift System (EARS). *P. boisei* has been characterized as an herbivorous C_4_ specialist, and paradoxically, its demise coincided with habitats favorable to its dietary ecology. Here we report new pedogenic carbonate stable carbon (δ^13^C_PC_) and oxygen (δ^18^O_PC_) values (nodules = 53, analyses = 95) from an under-sampled interval (1.4–0.7 Ma) in the Turkana Basin (Kenya), one of the most fossiliferous locales of *P. boisei*. We combined our new results with published δ^13^C_PC_ values from the EARS dated to 3–0 Ma, conducted time-series analysis of woody cover (ƒ_WC_), and compared the EARS ƒ_WC_ trends to regional and global paleo-environmental and -climatic datasets. Our results demonstrate that the long-term rise of C_4_ grasslands was punctuated by a transient but significant increase in C_3_ vegetation and warmer temperatures, coincident with the Mid-Pleistocene Transition (1.3–0.7 Ma) and implicating a short-term rise in *p*CO_2_. The contraction of C_4_ grasslands escalated dietary competition amongst the abundant C_4_-feeders, likely influencing *P. boisei*’s demise.

## Introduction

**Figure 1:**
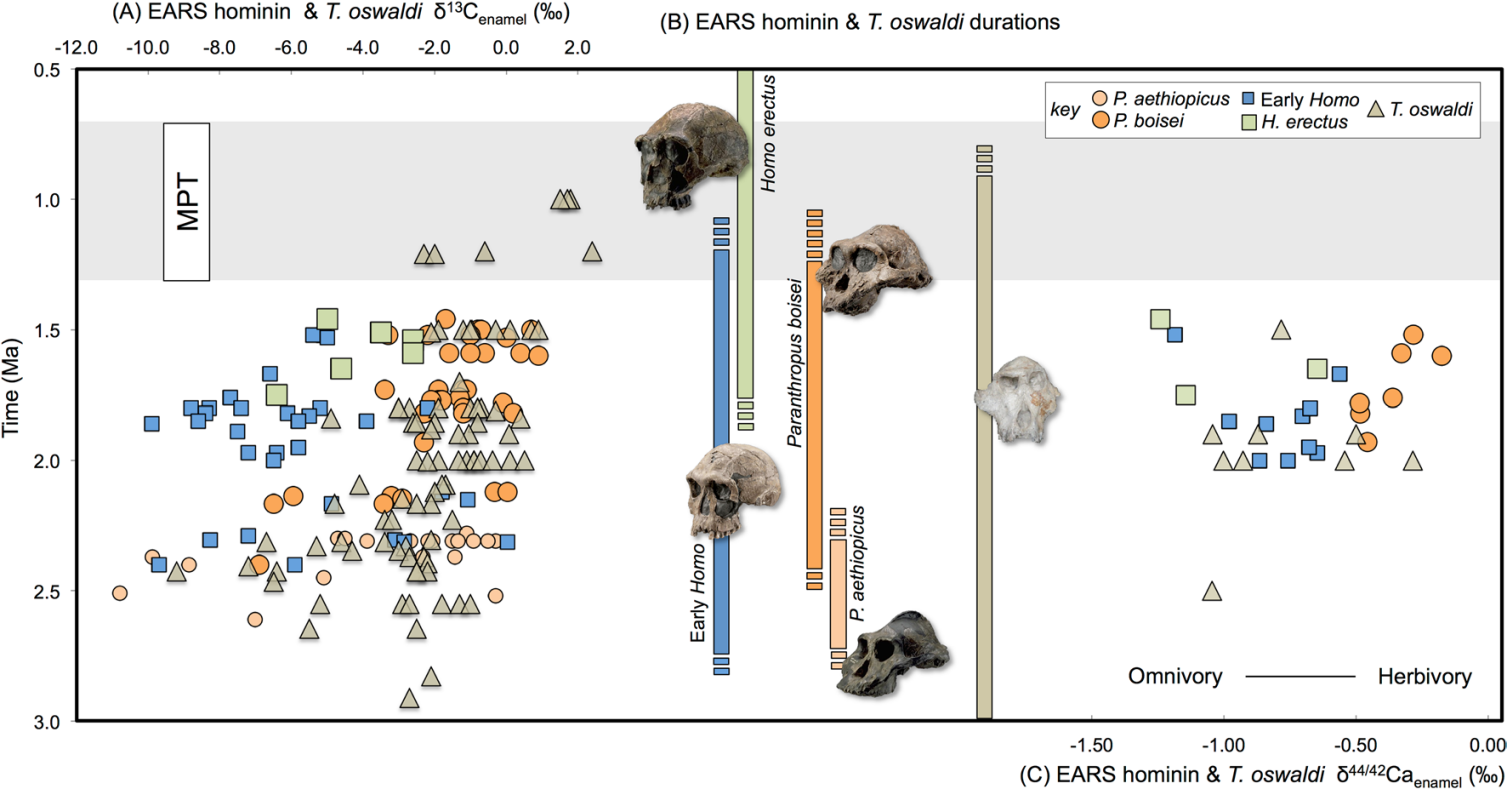
(**A**) δ^13^C_enamel_ values of EARS hominins and *Theropithecus oswaldi* (**B**) duration generalized taxonomic groupings of EARS fossil hominins, (**C**) δ^44/42^Ca_enamel_ values of EARS hominin and *T. oswaldi*. See Supplementary Data and Supplementary Information for specimen information, isotopic values, fossil image credits, and references. Shaded area denotes MPT interval (1.3–0.7 Ma).

Since Leakey and colleagues^1^ (pg. 9) suggested *Paranthropus boisei* was the “victim” of *Homo habilis* at Olduvai (Oldupai) Gorge (Tanzania), our ancestors have been implicated in the demise of their sister taxon. Tool-assisted foraging behaviors were traditionally thought to have propelled genus *Homo* into a broad omnivorous dietary niche, providing an evolutionary edge relative to *Paranthropus’* dentognathic procurement strategies across Africa’s savanna habitats^2^ and resulting in competitive exclusion^3^. More recent archaeological evidence suggests that both *Homo* and *Paranthropus* were plausible inheritors of tool-making behaviors^4,5^, and stable carbon isotopic (δ^13^C_enamel_) and microwear analyses of fossil specimens have revealed a dynamic and complex evolutionary history of Pleistocene African hominin and non-hominin primate diets (Fig. 1)^6–13^. Eastern African hominins and *Theropithecus*, the large-bodied baboon, underwent a dietary transition incorporating more C_4_ foods in the early Pleistocene^6,8,9,12^, which have been attributed to behavioral changes in response to complex competitive landscapes^12^. Distinct from other Pleistocene hominins, *P. boisei*, the last eastern African paranthropine species, yielded δ^13^C_enamel_ and δ^44/42^Ca_enamel_ values indicating a herbivorous^13^ and primarily C_4_ diet^6^ from ~ 2.3 Ma until its last appearance from the fossil record at ~ 1.3 Ma^14^. Unexpectedly, isotopic evidence for this shift into a C_4_-plant feeding niche was not mirrored by changes in microwear patterns or dentognathic morphology^7,10,15^. *P. boisei’s* diet had comparable mechanical properties to those of C_3_-C_4_-mixed feeding hominins such as *Australopithecus afarensis*^7^. *P. boisei’s* use of fallback foods, rather than its dietary staples of C_4_ plants, may have been the main influence for its distinctive “nut-cracking” form^7,10^.

Wood and Patterson (2020)^16^ suggested that *P. boisei’s* 1-myr morphological and dietary stasis signifies that its occupied niche and associated adaptations were “remarkably durable.” Although eastern African hominins may have begun their evolutionary trajectories as C_3_-C_4_-mixed-feeding opportunists during the early Pleistocene^11^, *P. boisei* evolved to be a C_4_ plant specialist^13^ sharing more dietary similarities to *T. oswaldi* than with members of genus *Homo*, who remained C_3_-C_4_-mixed feeding omnivores (Fig. 1A–C). Thus evidence for dietary niche separation and shared stone tool making abilities cast doubt on competitive exclusion by *Homo* as a primary cause of *P. boisei’s* demise.

A prevailing explanation for *P. boisei’s* extinction is the opposite cause of *Homo’*s success, that is, its dietary and behavioral *inflexibility* amidst environmental perturbations^17^. *P. boisei’s* extinction, estimated between 1.3 Ma^14^ and just prior to 1 Ma^18^, occurs during one of *Homo’s* significant increases in brain size and its second wave of dispersal out of Africa and into Eurasia^19,20^. These evolutionary events in the hominin lineage are coincident with the Mid-Pleistocene Transition (MPT; 1.3–0.7 Ma)^21^, when the Earth’s glacial and interglacial climatic intervals transitioned from an obliquity-paced (41-kyr) cyclicity to an asymmetrical ~ 100 kyr eccentricity pattern of repeated saw-toothed glacial growth and rapid deglaciation^22^. Global climatic and environmental changes are commonly evoked as major forces of eastern African hominin evolution^23^. Dietary and technological adaptations in the Plio-Pleistocene have been contextualized in open^24^ and variable^25^ landscapes, shaped by the presence of C_4_ plant communities^24^. During the MPT interval, current environmental proxy records from the East African Rift System (EARS) show evidence for low percentages of woody cover^24^ and high abundances of C_4_-feeders^26–29^. This scenario begs the question: why would *P. boisei*, this “durable” herbivorous C_4_-feeding hominin, disappear during the dominance of C_4_ grasslands?

**Figure 2:**
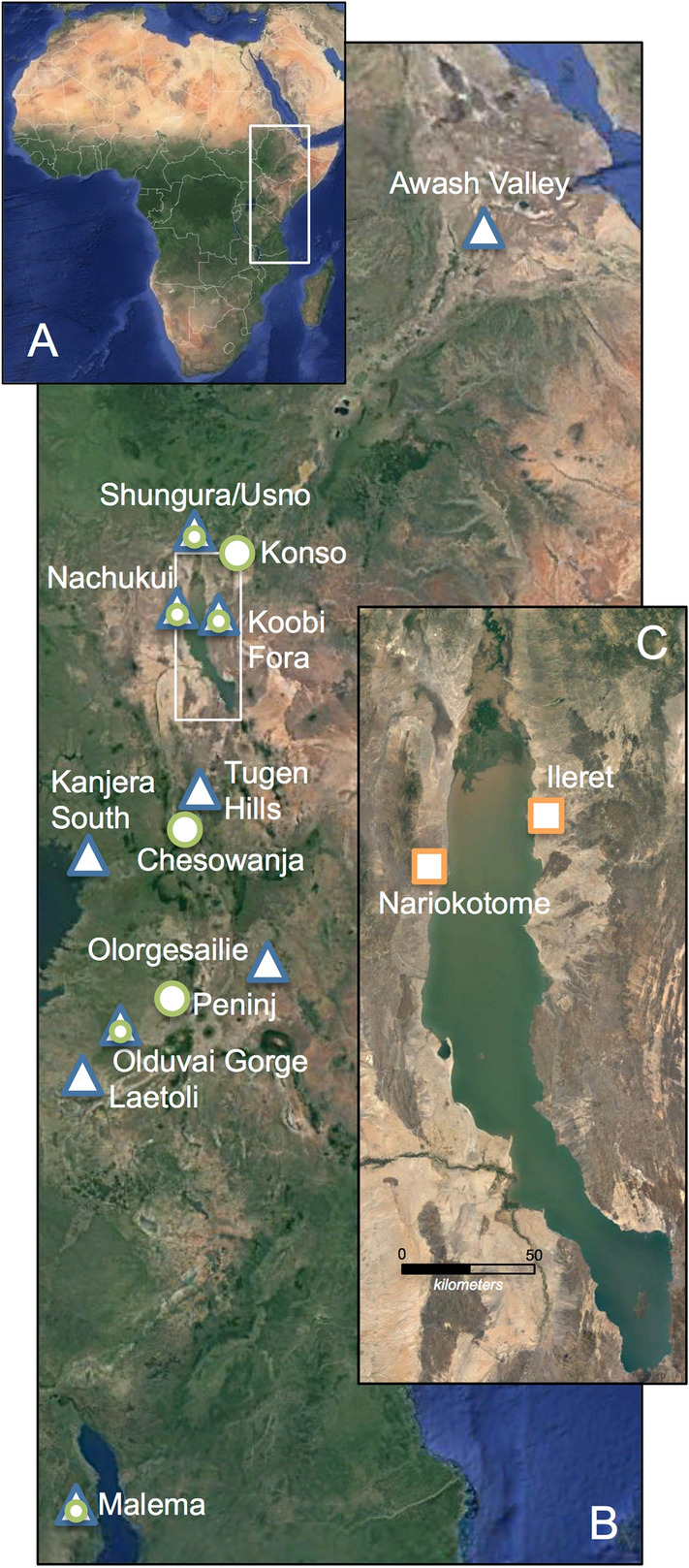
(**A**) Map of Africa with box outline showing (**B**) map of East African Rift System (EARS) with box outline showing (**C**) Turkana Basin (base map: Google Maps /TerraMetrics 2021). *P. boisei* fossil sites^18^ are denoted with a circle. Sampling locations for EARS ƒ_WC_ data that also preserved *P. boisei* specimens are marked with a circle embedded in a triangle. EARS ƒ_WC_ data from sites that did not yield *P. boisei* fossils are denoted with a triangle. Squares in inset map mark the locations of new δ^13^C_PC_ and δ^18^O_PC_ data presented in this study.

Here we report pedogenic carbonate stable carbon (δ^13^C_PC_) and oxygen (δ^18^O_PC_) isotopic values (nodules = 53, paired analyses = 95) in the Turkana Basin, northern Kenya, eastern Africa (Fig. 2A–C), from 1.4 to 0.7 Ma, an under-sampled interval for pedogenic carbonates in an otherwise well-studied region of human evolutionary environments (see "Methods", "Supplementary Information"). The Turkana region is the most fossiliferous of the known *P. boisei* sites^18^, and Turkana specimens constitute the majority of isotopically analyzed *P. boisei* enamel samples to date^6,8,12,13^. We combine our new δ^13^C_PC_ data to those from other EARS locations preserving the MPT interval, calculate fraction of woody canopy cover (ƒ_WC_)^24^, and compare the EARS ƒ_WC_ record to other environmental, ecological and climatic proxy records from 3 to 0 Ma to examine the contexts of *P. boisei’s* extinction.

## Results

**Figure 3:**
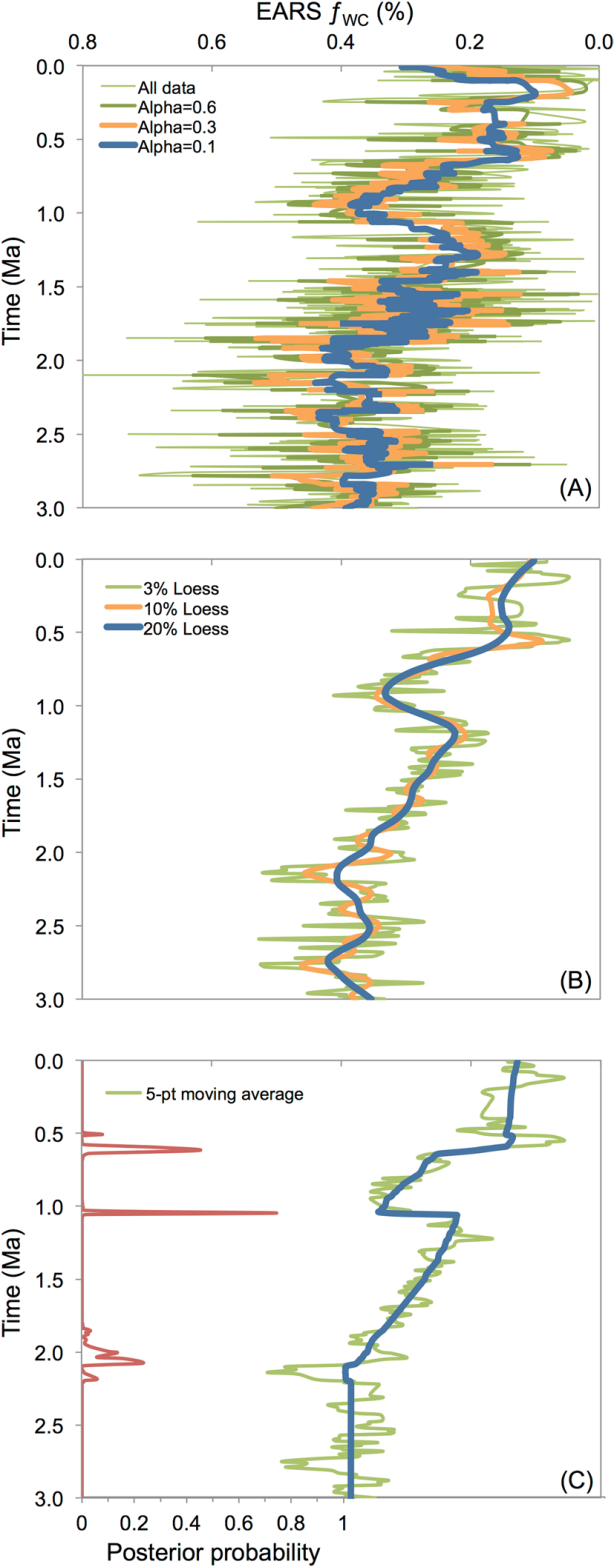
EARS ƒ_WC_ data fitted with (**A**) simple exponential smoothed curves (α = 0.1, 0.3, 0.6), (**B**) loess regressions (3%, 10%, 20%) and (**C**) Bayesian change point algorithm of a 5-point moving average; posterior probabilities on secondary axis (red line). See Supplementary Data for site locations, δ^13^C_PC_ values, and references. Shaded area denotes MPT interval (1.3–0.7 Ma).

In contrast to characterizations of the Turkana Basin during the MPT as extremely grassy and dry^23,24,28,29^, our new data (n = 53, 95 paired analyses) show excursions to lower δ^13^C_PC_ and δ^18^O_PC_ values, consistent with relatively woodier vegetation structures and more humid and/or warmer conditions (Supplementary Fig. S5). Exponentially smoothed ƒ_WC_ and δ^18^O_PC_ values from the Turkana Basin show temporally corresponding peaks of the excursions at 1 Ma (Supplementary Fig. S6). Exponential (Fig. 3A) and Loess (Fig. 3B) smoothing of the compiled EARS ƒ_WC_ record from 3 to 0 Ma illustrate the long-term increase in C_4_ vegetation punctuated by a short-term increase in C_3_ vegetation beginning at the start of the MPT interval and peaking at 1 Ma. The interpreted pattern from the EARS ƒ_WC_ record is substantiated with Bayesian change point analysis detecting two significant changes, which appear to mark the MPT interval (Fig. 3C).

African basins have idiosyncratic variables (e.g., elevation, topography, temperature, water deficits, tectonism) that influence the distribution of vegetation and local climatic conditions^23,30^. Individual EARS basins during the time of *P. boisei’s* evolutionary history show local-scale heterogeneity in vegetation structures (Supplementary Fig. S7). Some EARS sites have low δ^13^C_PC_ sampling resolutions or may be oversampled in specific time horizons. The Awash (Ethiopia) and Turkana (Kenya) basins have the highest δ^13^C_PC_ sampling resolutions across the MPT interval (1.3–0.7 Ma)^21^ with 27 and 53 samples, respectively, and both record significant reductions in C_4_ vegetation (Supplementary Fig. S7). Olduvai (Oldupai) Gorge and Tugen Hills, yielding small sample sizes from the MPT interval, i.e., 12 and 3, respectively, show persistent grassy vegetation structures (Supplementary Fig. S7). Differences in vegetation structures between the northern and central EARS may be an artifact of low sampling density in the central EARS or true spatial differences within the EARS.

## Discussion

### Causes of the EARS C_3_ excursion

**Figure 4:**
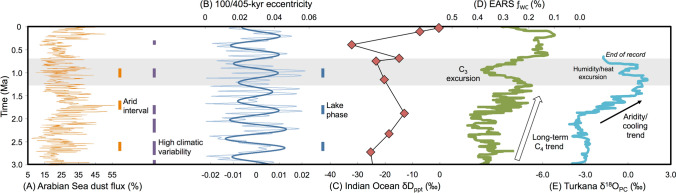
(**A**) Arabian Sea dust flux (%) from core sites 721/722 with proposed periods of increased aridity (orange bars)^35^ and heightened environmental variability^25^ (purple bars). (**B**) 100/405-kyr eccentricity record and proposed EARS lake phases^36^ (blue bars). (**C**) plant wax δD_ppt_ values from Indian Ocean core sites 235/241^38^, (**D**) EARS ƒ_WC_ record (exponentially smoothed, α = 0.1) (**E**) Turkana Basin δ^18^O_PC_ values. See Supplementary Data and Supplementary Information for site locations, δ^18^O_PC_ values, and references. Shaded area denotes MPT interval (1.3–0.7 Ma).

Vegetation structures are dependent on mean annual precipitation (MAP)^31^, and increasing abundances of C_3_ vegetation would be predicted with higher rainfall during the MPT. Several mechanisms are proposed to have altered water delivery to Plio-Pleistocene Africa, including eccentricity-modulated precession, glacial forcing of the Inter-Tropical Convergence Zone (ITCZ) position, and tropical sea-surface temperature fluctuations among others^33–38^, yet regional proxy records yield dissimilar evidence for hydroclimate (Fig. 4A–E). Dust flux data from marine cores at sites 721/722 in the Arabian Sea indicate several intervals of increased aridity, including one circa 1 Ma (Fig. 4A)^35^. In contrast, deep lakes in the EARS are thought to have formed during periods of higher rainfall and forced by 405-kyr eccentricity (insolation) maxima (Fig. 4B)^36^. During the MPT, Lake Silbo was present in the Turkana Basin^39,40^, possibly indicating higher rainfall.

Recent studies have suggested that hydroclimate may not be the primary cause of changes to African vegetation structures^38,41,42^. Plant wax δD values from ODP cores 235/241 in the Indian Ocean off of eastern Africa indicate no directional trend in calculated precipitation δD over the last 10 million years, and the MPT interval in those cores, specifically, does not appear to have experienced a significant shift in regional paleohydrology yielding intermediate values within the 3–0 Ma study interval (Fig. 4C)^38^. However, the sampling density (n = 3) of ODP 235/241 through the MPT is of particularly low resolution. If orbital climate forcing^36^ was the primary influence of the EARS C_3_ excursion, we would predict multiple C_3_ excursions coinciding with insolation maxima, which is not the case (Fig. 4D). The Turkana Basin δ^18^O_PC_ record, a proxy of rainfall source, rainfall amounts, and/or temperature, shows a long-term trend consistent with more arid and/or cooler conditions, followed by a return to warmer and/or wetter conditions during the MPT (Fig. 4E). Additional rainfall proxy records from the EARS, able to distinguish rainfall amount from sources of rain^42,43^, are warranted to further test for links between hydroclimate and vegetation structure.

**Figure 5:**
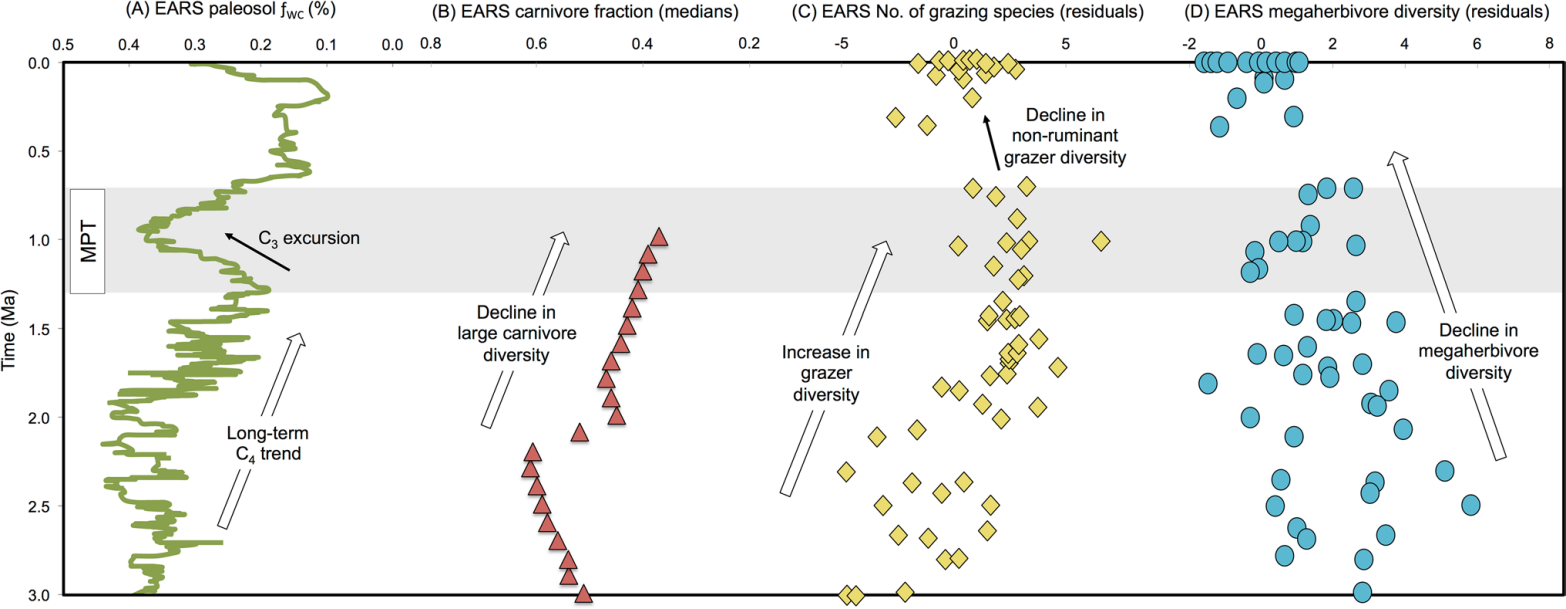
(**A**) EARS ƒ_WC_ record (exponentially smoothed, α = 0.1) compared to EARS fossil faunal abundance data: (**B**) carnivore fraction (medians);^44^ (**C**) number of grazing species (residuals);^45^ (**D**) megaherbivore diversity (residuals)^41^. Shaded area denotes MPT interval (1.3–0.7 Ma).

Vegetation structures are impacted by herbivore communities^31^; thus major changes in animal community compositions may have influenced the EARS C_3_ excursion (Fig. 5A–D). Significant declines in eastern African large-bodied carnivore speciosity from ~ 4–1 Ma (Fig. 5B)^44^ and megaherbivore diversity from ~ 6 to 0 Ma (Fig. 5D)^41^ correspond to the long-term EARS C_4_ trend (Fig. 5A). C_3_-browsing by fewer megaherbivores could have resulted in an increase in woody cover. However, after the C_3_ excursion, the EARS ƒ_WC_ record returns to previous percentages, maintaining the long-term C_4_ trend, which is not predicted as megaherbivores continued to decline. A recent compilation of the number of EARS grazer species from ~ 7 to 0 Ma^45^ parallels the spread of C_4_ grasslands as previous shown^23,26,28^; however, there is a major decline in the number of EARS grazer species, specifically non-ruminant grazers, beginning at ~ 1 Ma and coincident with the peak of the EARS C_3_ excursion (Fig. 5C). Faith and colleagues^45^ proposed that non-ruminant grazers were outcompeted by ruminant grazers and mixed feeders due to habitat loss during aridity pulses beginning with the MPT. The Turkana Basin δ^18^O_PC_ record is not consistent with increased aridity during the MPT, but the EARS ƒ_WC_ record indicates that C_4_ grasslands contracted significantly, which may have influenced the decline in non-ruminant grazers.

**Figure 6:**
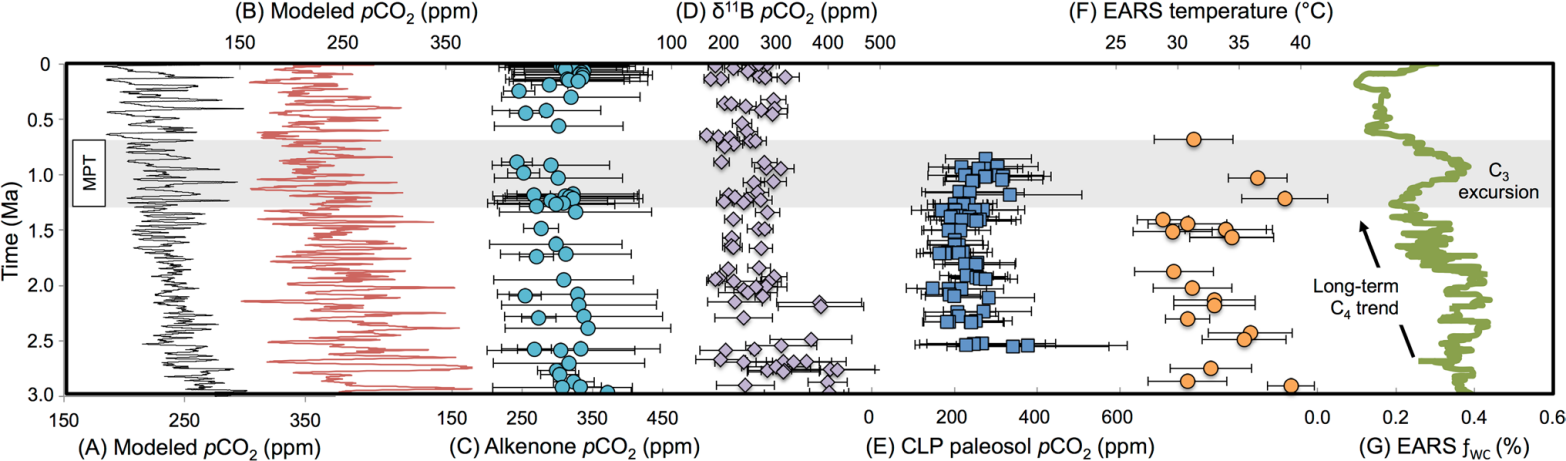
(**A**) Modeled global *p*CO_2_;^51^ (**B**) modeled global *p*CO_2_;^50^ estimated *p*CO_2_ (ppm) derived from compiled (**C**) phytoplankton-alkenone records^23^, (**D**) compiled δ^11^B values^23^, (**E**) Chinese Loess Plateau (CLP) paleosols^51^, (**F**) paleotemperature estimates based on clumped isotope analysis of EARS pedogenic carbonates^52^, (**G**) EARS ƒ_WC_ record (exponentially smoothed, α = 0.1). Shaded area denotes MPT interval (1.3–0.7 Ma).

The long-term increase in eastern African C_4_ grasslands and its impact on faunal communities has been associated to concurrently decreasing *p*CO_2_^38,41^. Global climate and vegetation models^46,47^ predict the cause-effect relationship between higher *p*CO_2_ and destabilized C_4_ vegetation^48^; moreover, woody thickening is proposed as a consequence of rising *p*CO_2_^46,47^. Modeled and proxy *p*CO_2_ records, showing discrepancies in estimations across the MPT (Fig. 6A–E), fuel various hypotheses about the role of *p*CO_2_ in Earth’s climatic reorganization^21,22,49–51^. The EARS ƒ_WC_ record (Fig. 6G) is consistent with the Chinese Loess Plateau paleosol *p*CO_2_ record^49^ (Fig. 6E) and one of the *p*CO_2_ models^51^ (Fig. 6A) indicating relatively higher *p*CO_2_ confined to the MPT interval. The clumped-isotope paleo-thermometer record, derived from paleosols in the Nachukui Formation at Turkana, shows higher temperatures during the MPT (Fig. 6F)^52^, which are predicted with increasing *p*CO_2_^53^. The Turkana Basin δ^18^O_PC_ excursion circa 1 Ma is also consistent with higher temperatures (Fig. 4E).

We interpret that the EARS C_3_ excursion was primarily forced by a transient increase in *p*CO_2_, potentially accompanied by an increase in temperature. There is debate, however, about the primary drivers of African vegetation change^38,54^, and vegetation proxy records from various African regions record dissimilar trends across the MPT. Plant wax δ^13^C data from ODP core 1077 in the Lower Congo Basin indicated an increase in C_4_ vegetation, which was interpreted as a response to an increased aridity circa 900 Ka^34^. Arabian Sea plant wax δ^13^C data (core sites 721/722) yielded evidence for a long-term increase in C_4_ vegetation but no significant change during the MPT interval^38^. Lake Malawi plant wax δ^13^C record, from the southern EARS (Malawi Rift), shows persistent C_3_ vegetation throughout the Plio-Pleistocene and across the MPT interval^55^. Our analysis of individual basins demonstrates that the C_3_ excursion occurred in the northern EARS and may not have been a significant event in the central EARS. However, we emphasize that sampling resolution of some of these records may not be sufficiently high to resolve vegetation changes within the MPT interval.

Finding discrepancies between the EARS ƒ_WC_ record and other regional vegetation datasets supports interpretations that marine sediments to the north and west of Africa may not capture the complete range of paleoenvironmental conditions within the EARS^56,57^. In contrast to the limited spatial averaging of pedogenic carbonates (see Supplementary Information), marine core records of terrestrial vegetation represent integrated signals without specific provenance for large regions, for example, aeolian transport from southern Africa in the case of ODP core 1077^34^. Site- and region-specific controls on vegetation may differentially respond to changes in ice volume, sea surface temperatures, and *p*CO_2_^38,57^. Moreover, model data and lake core studies demonstrate that rift basins respond differently to global environmental change such as during the Last Glacial Maximum^58,59^. Therefore, we suggest that a transient rise in global *p*CO_2_ causing the EARS C_3_ excursion does not necessitate synchronous declines in C_4_ vegetation in other African regions.

### A new behavioral and ecological scenario for the extinction of *P. boisei*

**Figure 7:**
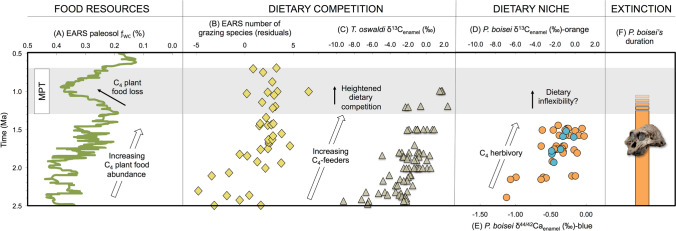
Proposed ecological and behavioral influences of *P. boisei’s* extinction. Food resources represented by (**A**) EARS ƒ_WC_ record (exponentially smoothed, α = 0.1); dietary competition shown with (**B**) speciosity of EARS grazers^45^ and (**C**) *T. oswaldi* δ^13^C_enamel_ values; dietary niche shown with (**D**) *P. boisei* δ^13^C_enamel_ and (**E**) δ^44/42^Ca_enamel_ values; estimated time of extinction denoted by (**F**) *P. boisei’s* duration^18^. See Supplementary Data and Supplementary Information for isotopic values, fossil image credit, and references. Shaded area denotes MPT interval (1.3–0.7 Ma).

*P. boisei’s* extinction occurred during a significant contraction of C_4_ grasslands within the EARS, and specifically in one of its known habitats, the Turkana Basin. Admittedly, ƒ_WC_ estimates do not fully characterize the diversity and complexities of African vegetation communities during the Pleistocene, but rather provide evidence for relative changes in the dominant (C_3_ vs. C_4_) vegetation structures. The decline in C_4_ grasslands likely resulted in the loss of *P. boisei’s* exploited C_4_ plant foods (Fig. 7A), but the identities of those C_4_ plant foods remain unknown. Microwear evidence suggest that foods items may not have involved hard components^7,10^ but rather “novel mechanical challenges” entailing masticating C_4_ grasses and sedges for long periods of time^12^. Faunal-based studies have emphasized that EARS environments during human evolution were “non-analogous” to modern faunal community structures^26,45,60^. In a common thread, paleovegetation communities also evolved through time, thus limiting the use of modern EARS environments and current vegetation proxy methods to identify specific elements of vegetation communities as well as particular plant species consumed by Pleistocene hominins. Moreover, dietary reconstructions of eastern African hominins and non-hominin primates with δ^13^C_enamel_ and δ^44/42^Ca_enamel_ data pose issues of isotopic equifinality^61^ where many types of food combinations may result in comparable isotopic values.

*P. boisei* likely competed directly with the EARS grazing species for C_4_ plant foods (Fig. 7B). We suggest that *P. boisei* and several other non-ruminant grazers were outcompeted by ruminant grazers for declining C_4_ plant foods during the MPT interval. Of course, *P. boisei* was not an herbivorous ungulate but rather a large-bodied, bipedal, encephalized, and likely stone tool using hominin^15,18,62^, thus its life history strategies, social organization, reproductive rates, activity times, caloric and nutritional requirements, home and day ranges, and cognitive abilities were likely more similar to other hominins and non-hominin primates than to most of the ungulates occupying the EARS C_4_ biome. *T. oswaldi* was likely in direct competition with *P. boisei* for C_4_ plant foods throughout the Pleistocene (Fig. 7C). δ^44/42^Ca_enamel_ values, however, suggest that *T. oswaldi* engaged in omnivory throughout its evolutionary history, providing some dietary niche separation from the herbivorous *P. boisei* but also a competitive advantage, as C_4_ plant foods contracted.

Although much evidence indicates dietary niche partitioning between *P. boisei* and members of *Homo*, some dietary competition may have occurred. Patterson and colleagues (2019)^63^ suggested that in the Koobi Fora region of the Turkana Basin after 1.65 Ma, *Homo* sp. δ^13^C_enamel_ values slightly converge with those of *P. boisei,* and *H. erectus* was by and large the C_4_ interloper (Fig. 1). The post-1.65 Ma change in *Homo* sp. δ^13^C_EC_ values was not detected in other sympatric mammals from the Koobi Fora region, and local vegetation structures appear stable; consequently, as these authors^63^ suggest, it seems likely that a behavioral change rather than a change in resource base underpinned *H. erectus’* dietary shift. *H. erectus* and other members of *Homo* display wide ranges of δ^44/42^Ca_enamel_ values after 1.65 Ma suggestive of omnivory (Fig. 1C). Ungulate C_4_-grazer meat and marrow may have been the primary source of *H. erectus’* C_4_ diet^13,63^, supporting the interpretation of niche separation rather than direct competition between the hominin sister taxa even after 1.65 Ma. But notably a few *Homo* specimens approach *P. boisei’s* δ^13^C_enamel_ and δ^44/42^Ca_enamel_ values^13^, which could be interpreted to indicate that segments of the omnivorous *Homo* populations exploited some resources within *P. boisei’s* dietary niche^12^.

Similar biogeographic distributions and habitat preferences of members of *Homo *and *P. boisei*^62^ implies competition for non-food resources including but not limited to feeding territories, sleeping sites, and potable water. Evidence for the origins^5,64^ and evolution^65,66^ of Early Stone Age technologies has shifted the discussion of presence vs. absence of tool making abilities by *P. boisei* and other non-*Homo* hominin species further toward the cognitive and social learning capacities required for habitual and advanced stone tool making^67^. The large-brained and -bodied *H. erectus* remains the most likely maker of advanced tools during the MPT interval^62^ and may have outcompeted * P. boisei* for non-food resources and some C_4_ plant foods with those tools.

In summary, EARS environments experienced a significant reduction in C_4_ grasslands during the MPT interval potentially forced by an increase in *p*CO_2_ and associated with a rise in temperature. The EARS C_3_ excursion, peaking circa 1 Ma, escalated dietary competition amongst the abundant C_4_-feeders, which influenced the decline of non-ruminant grazers. Dietary niche separation amongst the EARS hominins may have served as a strategy to reduce competition in the C_3_-C_4_-mixed feeding niche during the early Pleistocene. However, with C_4_ plant food loss, *P. boisei’s* inability to return to its ancestral C_3_-C_4_-mixed diet due to competitive exclusion by *H. erectus* and/or its own behavioral inflexibility likely played a role in its extinction (Fig. 7D–F).

## Methods

 Our study site is located in the Lake Turkana Basin, which is part of the northern Kenyan rift in the eastern branch of the EARS (Fig. 2A–C). On the northwest side of the basin, the Nariokotome Member is the uppermost unit of the Nachukui Formation and attains a thickness of ~ 60 m^69^. Previous interpretations suggested the Nariokotome Member was accumulated through the period of 1.30–0.75 Ma^70^. Near the Nariokotome Catholic mission, we studied this member’s outcrops and sampled pedogenic nodules along two NW trending transects (Supplementary Fig. S1, Supplementary Table T1). Two representative composite stratigraphic sections, measuring 50–55 m thick, were recorded (Supplementary Fig. S6). Walking along marker horizons (e.g., stromatolites layers) or using a transit to level the relative positions of marker horizons facilitated correlations between successive outcrops (Supplementary Fig. S2). Sedimentary strata of these outcrops have dips of about 3–5° into the west or are nearly flat lying. The strata comprise rounded volcanic-clast gravels, quartzo-feldspathic sands of varying grain sizes, and mudstones. Locally, the mudstones preserve carbonate nodules and slickensided fractures that indicate paleosols. Occasionally interbedded with these detrital clastic sediments are thinner units of stromatolite-encrusted gravels, mollusk sandstones, and tuff layers.

On the northeast side of the basin, the Koobi Fora Formation’s Chari Member (1.38–0.75 Ma) was examined because it is nearly time equivalent with the Nariokotome Member. We compiled fieldwork observations and samples from the Chari Member outcrops exposed near the town of Ileret (Supplementary Fig. S3). Sediments, chronostratigraphic constraints, and interpretations of the depositional environments for the Ileret outcrops have been described in detail elsewhere^39^. We sampled pedogenic carbonates from sedimentary strata documented by the section PNG-04^39^ The PNG-04 section is redrawn in Supplementary Figure S4 and relevant latitude and longitude data are listed in Supplementary Table T1. At PNG-04, an unconformity occurs ~ 7 m up from the base of the Chari Tuff. Samples were derived from stratigraphic levels above and below the unconformity, measured relative to the dated tuff units. Lithostratigraphic thicknesses, sedimentological data, and bedding attitudes were collected at the outcrops using standard field procedures and measuring instruments.

Pedogenic carbonate nodules were extracted from all preserved carbonate nodule-bearing paleosols throughout each of three outcrop sections in the Turkana Basin as the stratigraphic distribution of relevant geological materials dictated. Paleosols were identified from the presence of vertic features and slickensides. Pedogenic carbonate nodules were sampled at levels > 30 cm below the contact with overlying stratum and ~ 50 cm deep into the outcrop. Ages were determined through linear scaling between the stratigraphic levels of the radioisotopic dates of the Lower Nariokotome Tuff (1.30 Ma) and the Silbo Tuff (0.75 Ma) and the Chari Tuff (1.38 Ma) and the Silbo Tuff^70^. Scaled ages were calculated from the reported sedimentation rate^70^. The large age gap between samples Ileret 514–1 and 520–2 (Supplementary Fig. S5; Supplementary Data) is due to a ~ 500 kyr unconformity in the lower Chari Member (Supplementary Fig. S4)^39^.

Pedogenic nodules were cross-sectioned to expose the inner surface. Carbonate powders were eroded with a hand-held rotary tool (Foredom Series) affixed with a 0.5 mm carbide bit. We avoided sparry calcite and collected micrite from the nodules. Ninety-five δ^13^C analyses of extracted powders from fifty-three pedogenic carbonate (PC) nodules were conducted on a FISIONS Mass Spectrometer in the Department of Earth and Planetary Sciences at Rutgers University. Samples were reacted at 90 °C in 100% phosphoric acid for 13 min. δ^13^C_PC_ and δ^18^O_PC_ values are reported in the standard per mil (‰) notation: = (R_sample_/R_standard_ – 1)*1000, relative to Vienna-Pee Dee Belemnite (V-PDB) using the laboratory standard NBS-19. Analytical error is < 0.05‰.

We utilized δ^13^C_PC_ data from published sources and this study (n = 53) to characterize vegetation structures in each of the EARS basins dated to 3–0 Ma, which spans the evolutionary history of *P. boisei* (Supplementary Data). Published data were taken from the compilation of Levin (2015)^23^ and also from Quinn and others (2013)^71^, Patterson and colleagues (2019)^63^, and Potts et al. (2018)^72^. We then compiled δ^13^C_PC_ values from EARS sampling locations that included data spanning the MPT interval to gauge relative changes in vegetation structures from 3 to 0 Ma, which included Awash, Turkana, Olduvai Gorge, and Tugen Hills. Due to potentially different rainfall sources across different EARS basins^73^, we restricted time-series analysis to δ^18^O_PC_ data from the Turkana Basin and Lower Omo Valley.

After Cerling and others^24^ we subtracted 14‰ from the δ^13^C_PC_ values to convert to the isotopic equivalent of organic carbon (δ^13^C_*om*_) and used the equation: ƒ_WC_ = {sin[−1.06688 – 0.08538(δ^13^C_*om*_)]}^2^ to generate estimates of fraction woody canopy cover (ƒ_WC_) for classification into UNESCO categories of African vegetation (see Supplementary Information). Eastern African savanna plant communities demonstrate a wide range of δ^13^C_PC_ values, and due to differential paleosol deposition and preservation, δ^13^C_PC_ data points are not evenly distributed through time. In order to assess trends in the central tendency of vegetation structures through time from the EARS ƒ_WC_ datasets, we performed simple exponential smoothing (α = 0.1, 0.3, 0.6), Loess regressions (3%, 10%, 20%, 30%), and a Bayesian change point algorithm of a 5-point running mean (see Supplementary Information).

## Supplementary Information

Supplementary Information.

Supplementary Dataset.
