# Supplementary material for: Contracting eastern African C4 grasslands during the extinction of Paranthropus boisei
Source: Sci Rep. 2021 Mar 30;11:7164. doi: 10.1038/s41598-021-86642-z (PMC8009881; doi:10.1038/s41598-021-86642-z)
Supplement: Supplementary file 1 — Supplementary Information. [file 41598_2021_86642_MOESM1_ESM.pdf]

Supplementary Information for:

Contracting eastern African C_4 grasslands during the extinction of *Paranthropus boisei*

Quinn, R.L., Lepre, C.J.

This file includes:

Supplementary Information

A. Stable isotopic analyses of pedogenic carbonates for reconstructing vegetation structures and climatic conditions
B. Field sampling and age control
C. Data compilations and treatments
D. Turkana Basin and EARS pedogenic carbonate isotopic records
E. *P. boisei's* fossil sites and estimated time of extinction
F. EARS hominins and *T. oswaldi* specimen identification references and fossil image credits

Supplementary Figures S1-S7

Supplementary Table T1

Other supplementary information includes the following:

Supplementary Data

Supplementary Information

A. Stable isotopic analyses of pedogenic carbonates for reconstructing vegetation structures and climatic conditions

Woody vegetation (trees, shrubs), sedges and grasses utilizing the C_3 photosynthetic (Calvin-Benson) pathway discriminate against the heavier and kinetically slower isotope of carbon, ^{13}C; whereas grasses and sedges using the C_4 (Hatch-Slack) pathway allow greater incorporation of ^{13}C (1). Globally C_3 and C_4 plants show normal and non-overlapping distributions in $\delta^{13}C$ values (2,3). In eastern Africa, C_3 dicots from closed and open canopy forests have average $\delta^{13}C$ values of -31.4 ± 0.5‰ and -27.8 ± 0.3‰, respectively, and from savanna and bushlands, $\delta^{13}C$ averages of -27.0 ± 0.2‰ (4). Mesic C_4 grasses (NADP subpathway) have an average $\delta^{13}C$ value of -11.8 ± 0.2‰; xeric C_4 grasses utilizing the NAD and PCK subpathways have average $\delta^{13}C$ values of -13.1 ± 0.3‰ and -12.9 ± 0.1‰, respectively (4) in eastern African savanna environments.

Pedogenic carbonates at depths greater than 30 cm in soils with relatively high respiration rates incorporate CO_2 of decaying organic matter derived from surface vegetation during soil development (5-9). Vegetation-derived CO_2 $\delta^{13}C$ values are incorporated into soil organic matter (subscript SOM) and pedogenic carbonate nodules (subscript PC) preserved in paleosols, which are used to quantitatively measure the relative amounts of C_3 and C_4 biomass on the land surface during soil carbonate formation (10). Surface biomass CO_2 $\delta^{13}C$ values are generally comparable to $\delta^{13}C_{SOM}$ values (11), whereas $\delta^{13}C_{PC}$ values are enriched by +14-17‰ relative to biomass-derived CO_2 (9). Cerling and others (12) derived a regression equation to calculate the fraction of woody canopy cover (f_{WC}) based on measured $\delta^{13}C_{SOM}$ values of modern African and Australian soils, for categorizing habitat structure based on UNESCO classifications of African vegetation. Vegetation structures have been reconstructed from the many $\delta^{13}C_{PC}$-based studies of eastern African Plio-Pleistocene fossil hominin sites (12).

Stable oxygen isotopic values of pedogenic carbonates ($\delta^{18}O_{PC}$) are controlled by soil pore water $\delta^{18}O$ values, temperature-dependent isotopic fractionation during carbonate formation, evaporation rates and soil moisture (5, 12). At depths greater than 30 cm, soil pore water $\delta^{18}O$ values approximate those of meteoric water (5, 9, 14). Soil pore water $\delta^{18}O$ values can change from expected meteoric water $\delta^{18}O$ values due to evaporation and season of carbonate formation (9, 15). $\delta^{18}O_{PC}$ values are widely used to estimate rainfall source, soil moisture, and temperature in continental environments (16). However, Fox and others (17) caution that when utilizing $\delta^{18}O_{PC}$ to detect changes in temperature and aridity over geologic time it is impossible to deconvolve evaporative effects of soil water $\delta^{18}O$ and soil temperature changes without knowing either meteoric water $\delta^{18}O$ values or air/soil temperatures in the past. Moreover, changes in source rains can produce $\delta^{18}O_{PC}$ variations when temperature and rainfall amounts are constant (16-17).

There are several limitations with $\delta^{13}C_{PC}$ and $\delta^{18}O_{PC}$ methods for inferring

vegetation structure and climatic conditions. Obviously pedogenic carbonate isotopic analysis is limited to characterizing carbonate-bearing paleosols, which form with negative water budgets (5, 7, 18) and thus typically characterize environments that are relatively dry, at least seasonally. Fossil soils preserved in the Plio-Pleistocene Turkana Basin are dominated by paleo-Vertisols, formed under a dry season of four or more months and 250-1000 mm of annual moisture (19). The formation of pedogenic carbonate nodules is not common along perennial river channels due to ample water availability; moreover, soils tend to occur on stable land surfaces (18, 20-21). Pedogenic nodules primarily form in the warm season and differentially record times of soil dewatering (23-24) and therefore may underrepresent conditions during cool and wet seasons (25). Pedogenic nodule formation times also vary, averaging 10^1-10^3 of years (22) and therefore potentially dampen extreme variations in vegetation structure with changing paleohydrological and paleogeography (e.g., river channels migrate laterally across the landscape and/or lake margins transgress and regress) as well as with seasonal changes. Pedogenic carbonate $\delta^{13}C_{PC}$ reflect the vegetation and soil water conditions of less than a square meter during nodule formation (5, 26). Sampling paleosols laterally yields variability in $\delta^{13}C_{PC}$ values (27-30) as would be expected in African savanna ecosystems (19, 27) with transitioning vegetation structures influenced by the local hydrological configuration, geography, and geomorphology as well as regional and global factors such as $p\mathrm{CO}_2$, rainfall and temperature. Sampling a small spatial scale may over-represent microenvironments due to limited paleosol preservation.

B. Field sampling and age control

At the outcrop (Supplementary Figures S1-S4), we identified pedogenic carbonate nodules within paleosols by criteria of Retallack (31) and applied to the Turkana Basin by Wynn (19, 27). We sampled pedogenic carbonates in the preserved calcic horizon of the paleosol at a minimum of 30 cm below the contact with the overlying stratum (7) and excavated back from the vertically exposed surface by approximately 50 cm. Calcite nodules were extracted from within individual peds. Since most Turkana fossil soils are paleo-Vertisols, they show vertic features and slickensided surfaces; we chose calcite nodules that exhibited slickensides and/or were adjacent to slickenslided surfaces. Although abundant in the formation deposits, we did not include calcareous rhizoliths in this study due to isotopic alteration by shallow cementation (32) and groundwater (33).

Age control of pedogenic carbonate samples was determined with the established chronostratigraphic framework and scaled with linear sedimentation relative to dated tuffs (34-35). The Brunhes-Matuyama Boundary occurs within the Chari Member approximately 5 m below the stratigraphic level of the Silbo Tuff (36) and was further used to scale collected samples relative to the ~500-kyr depositional hiatus (37). We utilized the date of 0.77 Ma for the Brunhes-Matuyama Boundary (38).

C. Data compilations and treatments

We combined our new $\delta^{13}C_{PC}$ and $\delta^{18}O_{PC}$ values from Nariokotome and Ileret to those reported in the Turkana Basin from the Nachukui and Koobi Fora Formations, respectively, from 3-0 Ma (data from 19, 27-30, 40-41). Utilizing methods of Cerling and others (12), we subtracted 14‰ from the $\delta^{13}C_{PC}$ values to convert to the isotopic equivalent of organic carbon ($\delta^{13}C_{om}$) and used the equation: $f_{WC} = \{\sin[-1.06688 - 0.08538(\delta^{13}C_{om})]\}^2$ to generate estimates of fraction woody canopy cover for classification into UNESCO categories of African vegetation. These categories were taken from White (42) and have the following $\delta^{13}C_{PC}$ value ranges of pedogenic carbonates (12): 1) forest: continuous stand of trees at least 10-m tall with interlocking crowns ($\delta^{13}C_{PC}$: >-11.5‰), 2) woodland/bushland/shrubland: woodland is an open stand of trees at least 8-m tall with woody cover exceeding 40% and a field layer dominated by grasses; bushland is an open stand of bushes usually between 3- and 8-m tall with woody cover exceeding 40%; and shrubland is an open or closed stand of shrubs up to 2-m tall ($\delta^{13}C_{PC}$: -11.5 to -6.5‰), 3) wooded grassland: land covered with grassland and has 10-40% tree or shrub cover($\delta^{13}C_{PC}$: -6.5 to -2.3‰), and 4) grassland: land covered with herbaceous plants with less than 10% tree and shrub cover ($\delta^{13}C_{PC}$: <-2.3‰).

Plio-Pleistocene eastern African environments preserve evidence for mosaic vegetation structures and habitat heterogeneity across relatively small spatial extents. Compilations of $\delta^{13}C_{PC}$ values show large ranges (43-44) especially for those locations that were sampled across synchronous units (28, 45-47). $\delta^{18}O_{PC}$ are also highly variable in eastern African environments (30). We analyzed f_{wc} trends for each sampling location and/or geologic formation with simple exponential smoothing (α=0.1) to dampen extreme variations and determine central tendency shifts in the woody cover during the evolutionary history of *P. boisei*. Locations/geologic formations that have been sampled for $\delta^{13}C_{PC}$ analysis (*Supplementary Data*) include the Hadar and Busidima formations in the Awash Basin (Ethiopia), the Nachukui and Koobi Fora formations in the Turkana Basin of northern Kenya, the Shungura Formation in the Lower Omo River Valley in southern Ethiopia, at Olduvai (Oldupai) Gorge in Tanzania, in the Tugen Hills region of central Kenya, at Kanjera South on the shores of Lake Victoria in western Kenya, at Olorgesailie in central Kenya, and in Karonga Formation of Malawi.

We compiled data from locations that provided a record across the MPT interval in order to detect relative changes in vegetation structures and to minimize impacts from oversampled single locations and intervals. These included the Awash Basin, the Turkana Basin, Tugen Hills, and Olduvai (Oldupai) Gorge. Simple exponential smoothing (α=0.1, 0.3, 0.6) and Loess (locally estimated scatterplot smoothing) (48) were applied to the compiled EARS f_{WC} record to estimate changes in the central tendency of vegetation structures through time. We utilized the Bayesian change point algorithm (49) of a 5-point running mean of the EARS f_{WC} record to detect significant changes in the long-term C_4 trend. Probabilities (posterior) of a change point being selected from the model were generated to detect significance (49). We compared three different

methods of time-series analysis to detect temporal changes in the f_{WC} record due to the wide data scatter and differential sampling resolution. Due to differences in rainfall across the EARS (50) and current $\delta^{18}O_{PC}$ data availability, we limited the $\delta^{18}O_{PC}$ data compilation and simple exponential smoothing (α=0.1) methods to those from the Turkana Basin. Statistical analyses were conducted with Acycle v2.2.

D. Turkana Basin and EARS pedogenic carbonate isotopic records

$\delta^{13}C_{PC}$ values (n=53, 95 analyses) averaged -5.2 ± 1.7‰. These results indicate an average fraction of woody canopy cover (f_{WC}) of 30%, ranging from 4-62% (Supplementary Fig. S5). Vegetation structural categories present during the MPT include woodlands, grassy woodlands, wooded grasslands, and grasslands. Forests are not indicated with these data. $\delta^{18}O_{PC}$ values (n=53, 95 analyses) have an average of 0.3 ± 0.4‰ (Supplementary Fig. S5). The majority of the MPT interval data points reported here are derived from the Nachukui Formation due to differential preservation (*Supplementary Data*). At Ileret, the 500-kyr depositional hiatus in the Koobi Fora Formation (37) does not capture the full record of vegetation structure during the MPT interval (Supplementary Fig. S5). The Shungura Formation in the Lower Omo Valley to the north of the Turkana Basin shows a trend toward relatively lower $\delta^{13}C_{PC}$ and $\delta^{18}O_{PC}$ values at the start of the MPT interval but has not been sampled later than 1.2 Ma (Supplementary Fig. S7) (30). Notably, the two parallel sections spaced ~1 km apart at Nariokotome demonstrate comparable $\delta^{13}C_{PC}$ and $\delta^{18}O_{PC}$ values (Supplementary Fig. 4), and both show excursions to lower values at 1 Ma (Supplementary Fig. S5).

The three coeval geologic formations of the Turkana Basin (Koobi Fora, Nachukui) and Lower Omo River Valley (Shungura) preserve differential depositional settings through time due to a number of factors including regional tectonic events and basin infilling, evolving hydrological configuration, and geographic and geomorphologic differences across subregions, amongst others (51-52). Differences in depositional setting have been shown to influence $\delta^{13}C_{PC}$ values in the Plio-Pleistocene EARS (28, 30). Lacustrine environments as preserved in the Nachukui and Koobi Fora formations had 10-20% less f_{WC} than those in riverine environments in the Shungura (Supplementary Fig. S7). We collected pedogenic carbonate samples from alluvial paleosols found adjacent to the margin of paleo-Lake Silbo (37,51) Our finding of relatively more C_3 vegetation on the margins of paleo-Lake Silbo is the opposite of predicted by depositional setting (28, 30).

Individual EARS basins show a wide range of f_{WC} estimates (Supplementary Fig. S7) and also differential sampling resolution. For example, Malawi f_{WC} record (53) near the early Pleistocene *P. boisei* site of Malema show evidence for persistent C_3 vegetation from the late Pliocene to the middle Pleistocene, yielding f_{WC} estimates between 40-60%, but the record lacks the MPT interval entirely. The Shungura Formation preserving many *P. boisei* fossils shows a fluctuating trend through time but is lacking the majority of the MPT interval. Two subsets of the compiled EARS f_{WC} record from Olorgesailie and

Kanjera South yield evidence for pure grasslands but are potentially oversampled in single locales and time horizons. Olorgesailie was sampled may times across one paleosol/time horizon dated to 0.99 Ma (n=61) (45) and may have included phreatic components according to additional analyses reported by Levin in the Supplementary Information of Potts and others (54). Kanjera South on the Homa Peninsula was sampled many times across one paleosol/time horizon dated to 2.0 Ma (n=22) (55). Neither of these locations offers sampling resolutions for relative changes in vegetation structures through time.

The three different time-series analyses of the compiled EARS record all yield excursion peaks at 1 Ma toward C_3-dominated vegetation structures. Based on the three generated curves, the EARS C_3 excursion equates to between a 10% and 20% reduction in woody cover within wooded grasslands. The posterior probability of the change point at 1 Ma exceeds 70%. Exponentially smoothed $\delta^{18}O_{PC}$ record spanning 3-0 Ma from the Turkana Basin and Lower Omo Valley shows a increasing trend toward higher $\delta^{18}O_{PC}$ values from ~2.2-1.2 Ma, potentially indicating increased aridity and/or decreasing temperature, before the excursion back to lower $\delta^{18}O_{PC}$ values at 1 Ma (Figure 5e).

E. P. boisei's *fossil sites and estimated time of extinction*

P. boisei fossils have been discovered throughout the EARS (Fig. 1) (56). Fossil sites that have also been sampled for $\delta^{13}C_{PC}$ include the Turkana Basin (Nachukui and Koobi Fora formations) of northern Kenya, the Lower Omo River Valley (Shungura Formation) in southern Ethiopia, Olduvai (Oldupai) Gorge in Tanzania, the Chesowanja site near the Tugen Hills, and the Karonga Formation at the site of Malema in Malawi. Although no $\delta^{13}C_{PC}$ values are yet reported, *P. boisei* has also been recovered from Konso and Peninj. *P. boisei* fossils have not yet been discovered in the Awash Basin, Kanjera South, or Olorgesailie, but those sites have been sampled for $\delta^{13}C_{PC}$ analysis.

The discovery of the *P. boisei* partial skeleton, OH-80, at Olduvai (Oldupai) Gorge in Tanzania provides its current last appearance datum of 1.34 Ma (57). The LAD is not likely the exact time of extinction (58), but it provides a lower limit of the extinction interval, which may be significantly later in time due to taphonomic factors (59). *P. boisei's* time of extinction is estimated as "at some point before 1.0 Ma" (56, pg. 109) and more recently as "just over 1 Ma" (60, pg. 23202). We use the interval of ~1.3-1.0 Ma as the time of *P. boisei's* extinction.

F. EARS hominins and T. oswaldi *specimen identification references and fossil image credits*

Dietary isotopic values shown in Fig. 1 and listed in Supplementary Data include only those reported to species-level designations for *Paranthropus* (*P. aethiopicus*, *P. boisei*) and *Theropithecus (T. oswaldi)*. Genus *Homo* specimens were separated into two groupings, Early *Homo* and *H. erectus*, after (61). Early *Homo* includes specimens designated as *H. habilis*, *H. rudolfensis*, or *Homo* indet. Photo thumbnails of hominin skulls shown in Figures 1 and 7 courtesy of the Human Origins Program, Smithsonian Institution; photo thumbnail of *T. oswaldi* skull (62) courtesy of Stephen Frost.

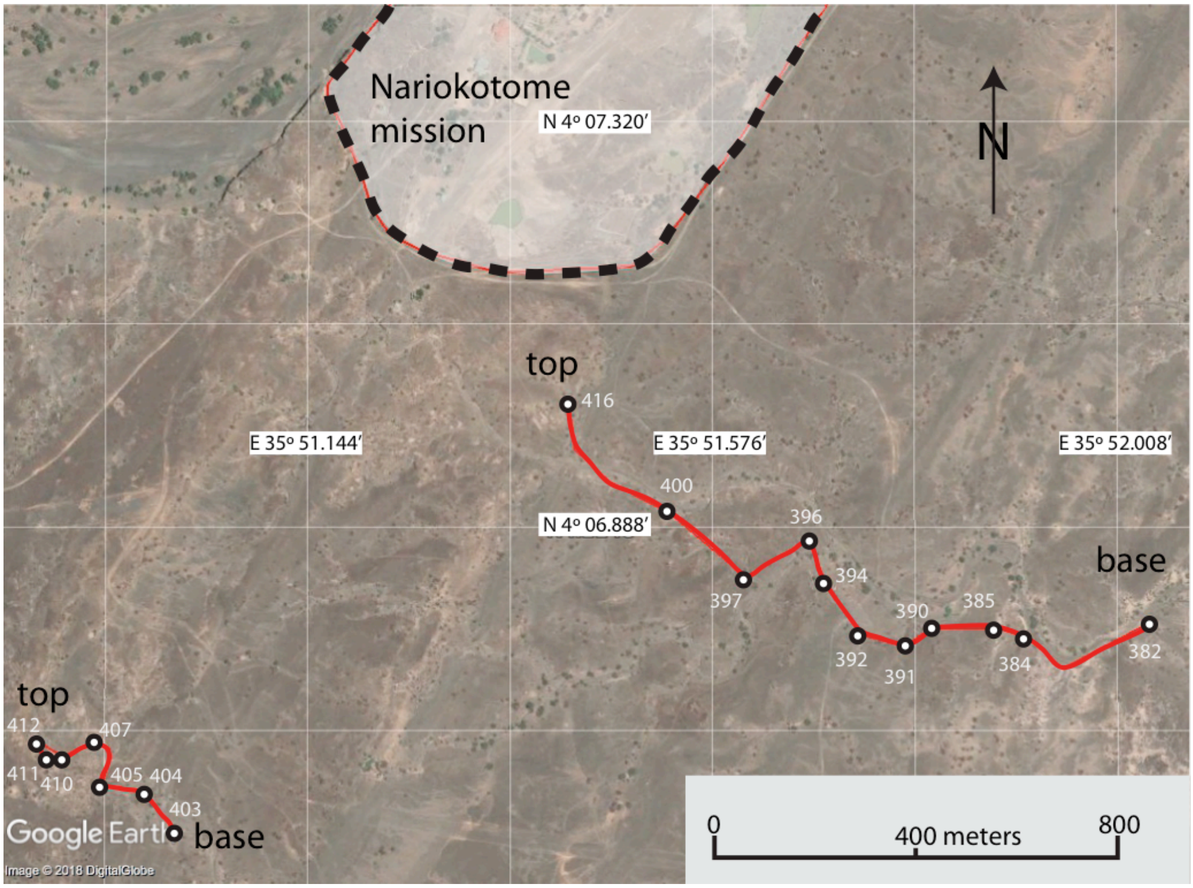

Supplementary Figure S1. Location of the Nariokotome Member study area, northwest Turkana Basin, northwest Kenya (base map: Google Maps/ TerraMetrics 2021). Red lines indicate transects through the outcrops sampled for pedogenic carbonates. See Supplementary Figure S2 for composite stratigraphic sections representative of outcrops. Supplementary Table T1 provides latitude and longitude for numbered points along transects.

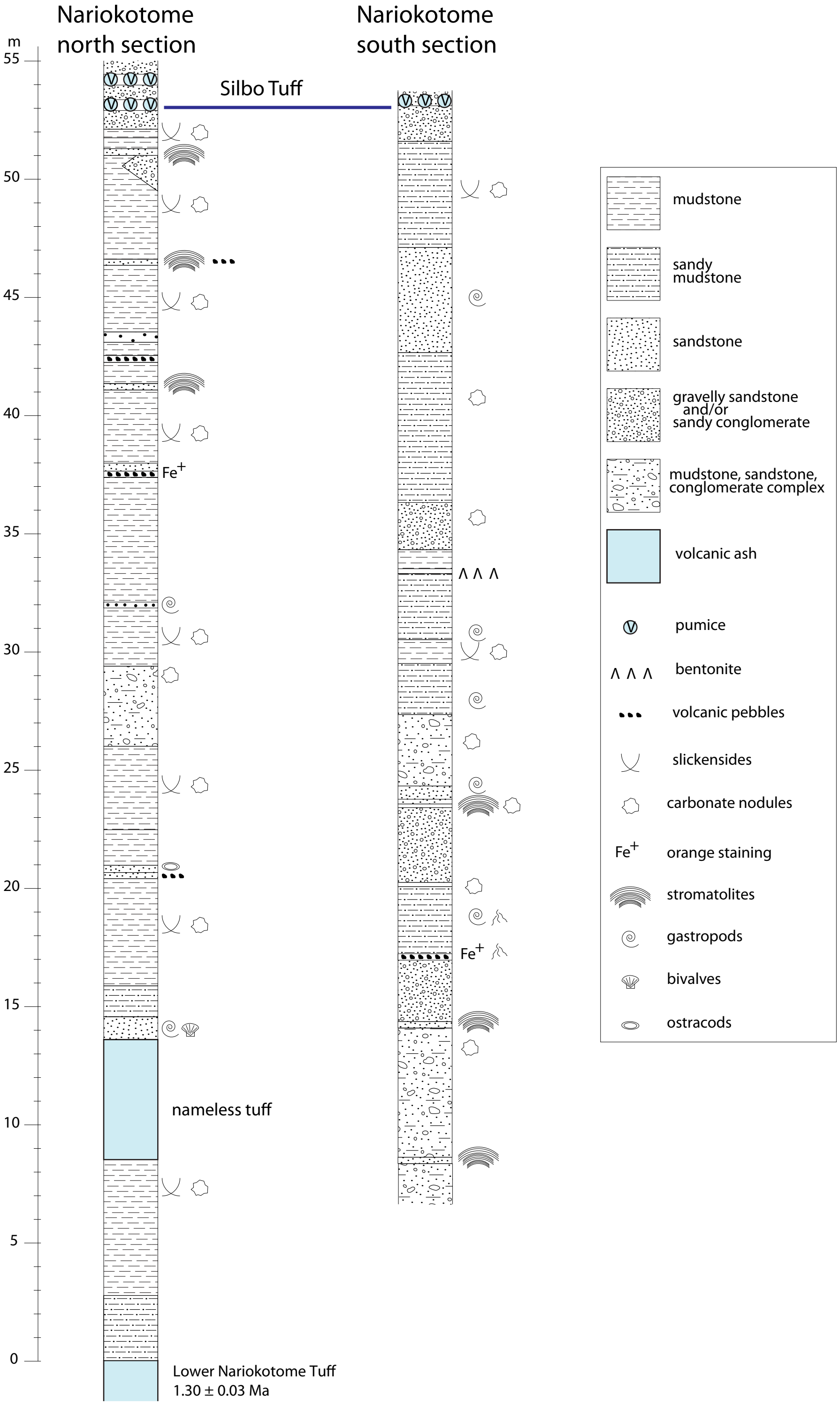

Supplementary Figure S2. Composite stratigraphic sections of Nariokotome sediments (1.30-0.75 Ma).

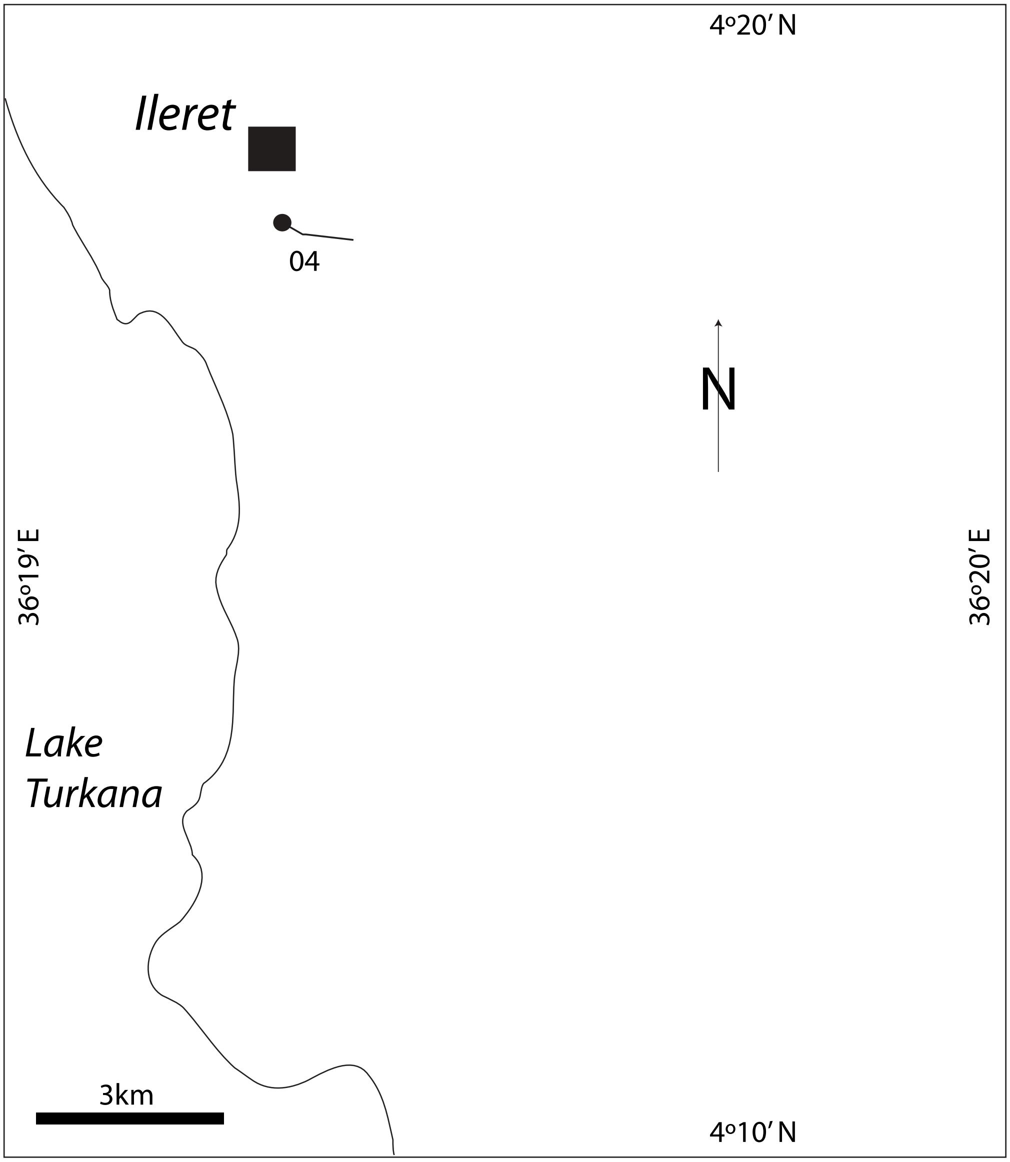

Supplementary Figure S3. Location of the Chari Member study area, northeast Turkana Basin, northwest Kenya. Map shows the location of stratigraphic section (Supplementary Figure S4) relative to the town of Ileret and the outline of the lakeshore.[21]

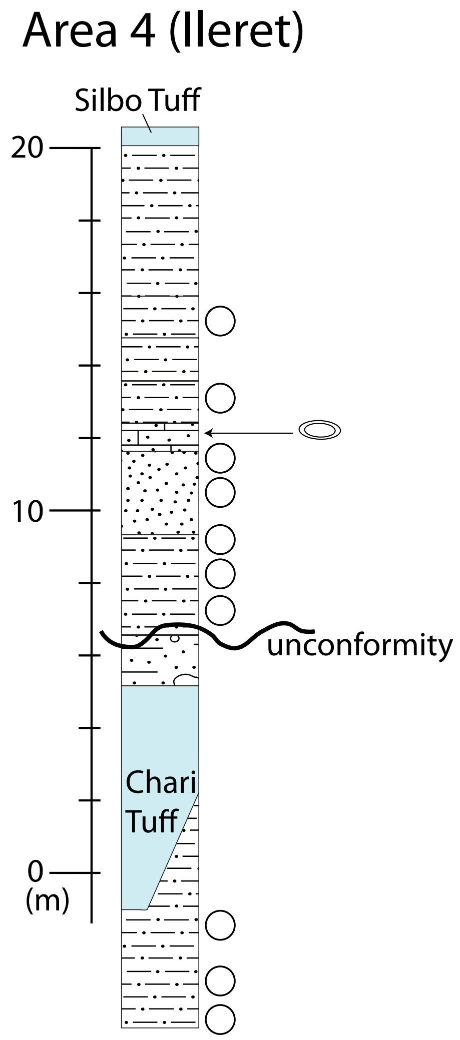

Supplementary Figure S4. Composite stratigraphic section of the Chari Member (1.38-0.75 Ma) from Area 4 of Ileret. Symbols denote ostracod marker horizon (oval)[15] and pedogenic carbonate samples collected for this study (open circles). See Supplementary Figure S2 for key.

Northeast transect at Nariokotome	
Mark	Position
382	N4° 06.785' E35° 52.044'
384	N4° 06.771' E35° 51.910'
385	N4° 06.780' E35° 51.879'
390	N4° 06.781' E35° 51.810'
391	N4° 06.762' E35° 51.783'
392	N4° 06.774' E35° 51.732'
394	N4° 06.830' E35° 51.695'
396	N4° 06.875' E35° 51.681'
397	N4° 06.833' E35° 51.611'
400	N4° 06.906' E35° 51.528'
416	N4° 07.020' E35° 51.423'
Southwest transect at Nariokotome	
Mark	Position
403	N4° 06.565' E35° 51.004'
404	N4° 06.606' E35° 50.971'
405	N4° 06.613' E35° 50.925'
407	N4° 06.661' E35° 50.916'
410	N4° 06.642' E35° 50.883'
411	N4° 06.644' E35° 50.867'
412	N4° 06.659' E35° 50.858'
Area 4 section at Ileret (Gathogo & Brown, 2006)	
Base sec. 4	N4° 18.090' E36° 14.058'

Supplementary Table T1. Latitude and longitude for numbered points along transects.

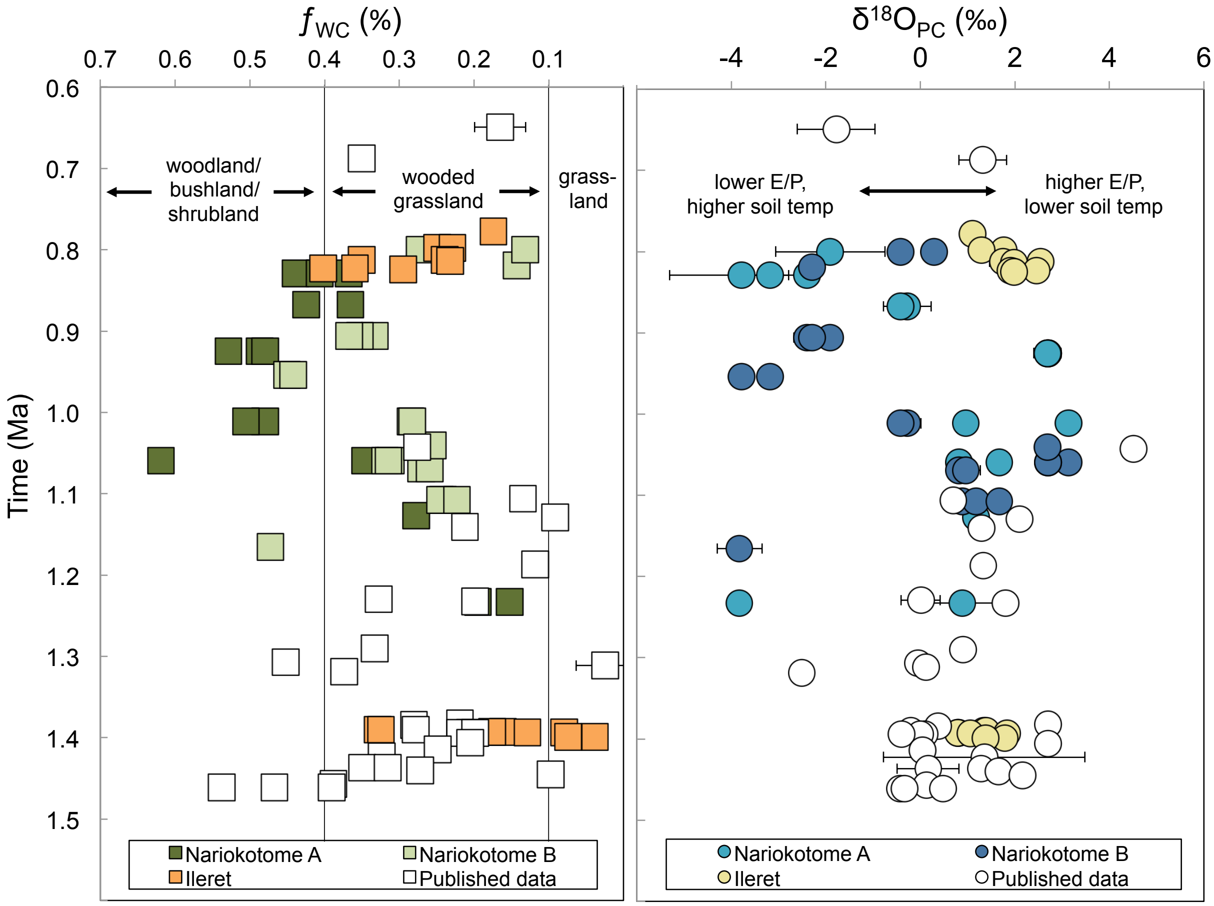

Supplementary Figure S5. New f_{WC} estimates and $\delta^{18}O_{PC}$ values dated to 1.4-0.7 Ma from the Turkana Basin generated in this study. All $\delta^{13}C_{PC}$ and $\delta^{18}O_{PC}$ values and f_{WC} estimates are provided in *Supplementary Data*.

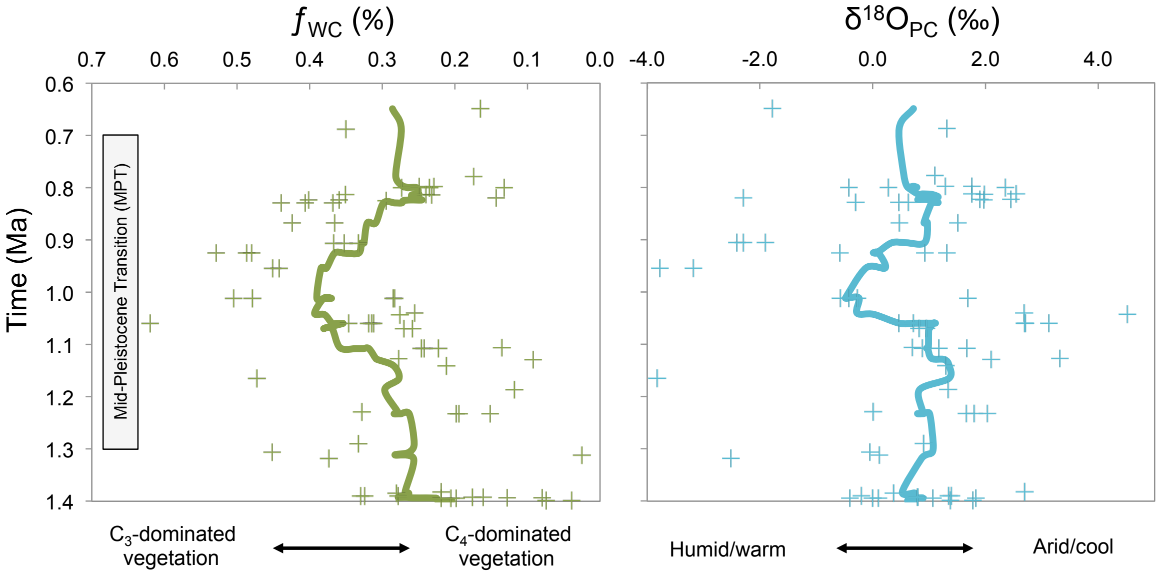

Supplementary Figure S6. Turkana Basin f_{WC} estimates and $\delta^{18}O_{PC}$ values during the Mid-Pleistocene Transition (MPT) interval (1.3-0.7 Ma) each fitted with an exponentially smoothed curve (α = 0.1). $\delta^{13}C_{PC}$ and $\delta^{18}O_{PC}$ values, f_{WC} estimates, and data references are listed in *Supplementary Data*.

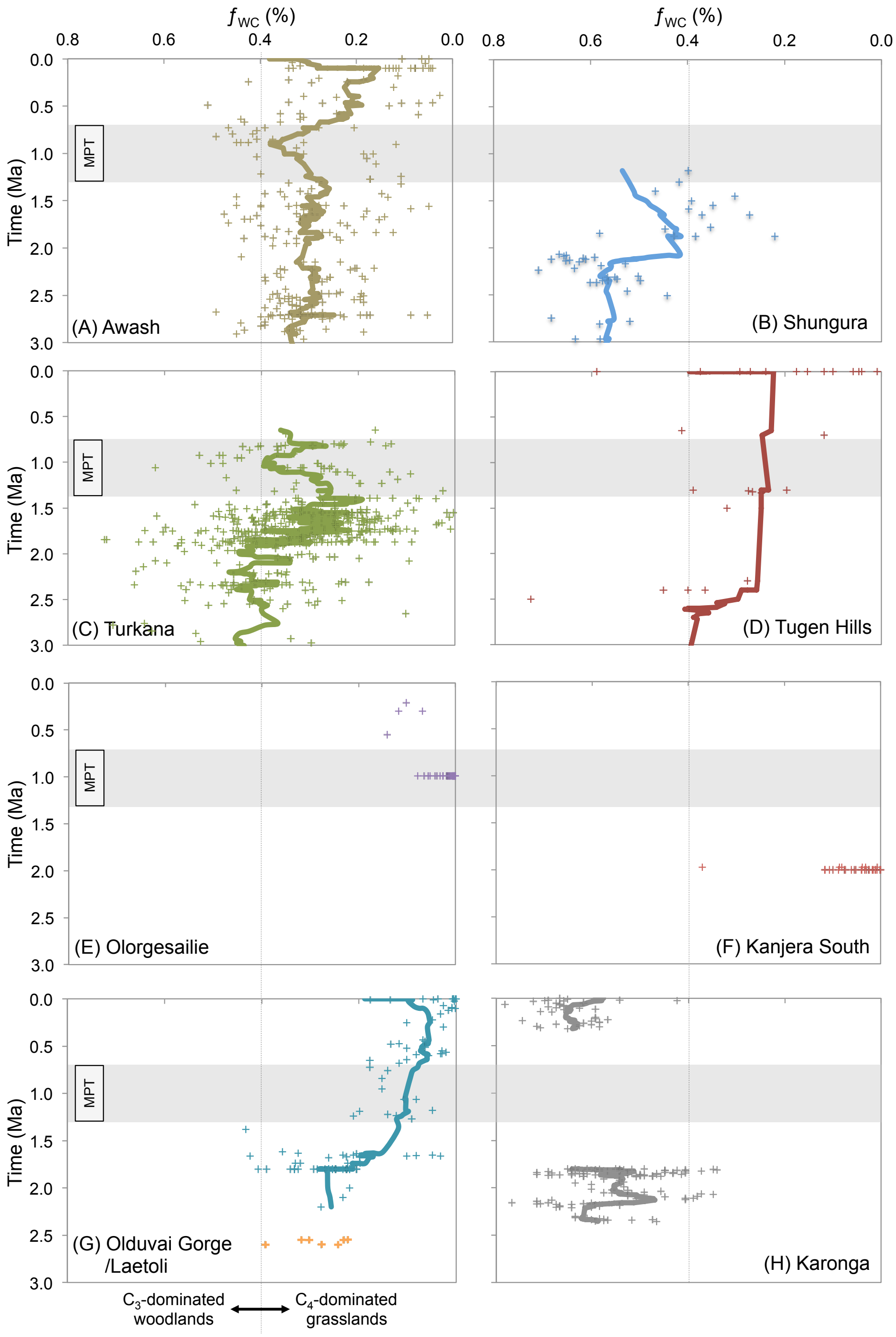

Supplementary Figure S7. f_{WC} data points from 3-0 Ma fitted with an exponentially smoothed curve (α = 0.1) of individual African basins. MPT interval is denoted with gray shaded band. Line demarcates f_{WC} = 40%, which separates C_3- and C_4-dominated vegetation structures. $\delta^{13}C_{PC}$ values, f_{WC} estimates, and data references are listed in *Supplementary Data*.

G. References

1. Tieszen, L.L., 1991. Natural variation in the carbon isotope values of plants: implications for archaeology, ecology, and paleoecology. Journal of Archaeological Science 18, 227-248.
2. Kohn, M.J., 2010. Carbon isotope compositions of terrestrial C_3 plants as indicators of (paleo)ecology and (paleo)climate. Proceedings of the National Academy of Sciences of the United States of America, 107, 19691-19695.
3. Sage, R.F., Monson, R.K., 1999. C_4 plant biology. San Diego, CA: Academic.
4. Cerling, T.E. et al., 2003. Diets of East African Bovidae based on stable isotope analysis. Journal of Mammalogy 84, 456-470.
5. Cerling, T.E., 1984. The stable isotopic composition of modern soil carbonate and its relationship to climate. Earth and Planetary Science Letters 71, 229-240.
6. Cerling, T.E. et al., 1989. Carbon isotopes in soils and palaeosols as ecology and palaeoecology indicators. Nature, 341, 138-139.
7. Quade, J. et al., 1989. Systematic variations in the carbon and oxygen isotopic composition of pedogenic carbonate along elevation transects in the southern Great Basin, United States. Geological Society of America Bulletin 101, 464-475.
8. Cerling, T.E. et al., 1991. On the isotopic composition of carbon in soil carbon dioxide. Geochimica et Cosmochimica Acta, 55, 3403-3405.
9. Cerling, T.E., Quade, J., 1993. Stable carbon and oxygen isotopes in soil carbonates. AGU Geophysical Monographs 78, 217-231.
10. Cerling, T.E., 1992. Development of grasslands and savannas in East Africa during the Neogene. Palaeogeography, Palaeoclimatology, Palaeoecology 97, 241-247.
11. Tieszen, L.L. et al., 1997. NDVI, C-3 and C-4 production and distributions in Great Plains grassland land cover classes. Ecological Applications, 7, 59-78.
12. Cerling, T.E. et al., 2011. Woody cover and hominin environments in the past 6 million years. Nature 476, 51-56.
13. Kim, S.-T., O'Neil, J.R., 1997. Equilibrium and nonequilibrium oxygen isotope effects in synthetic carbonates. Geochimica et Cosmochimica Acta 61, 3461-3475.
14. Amundson, R.G., Wang, Y., 1996. The relationship between the oxygen isotopic composition of soil CO_2 and soil water. IAEA, Isotopes in Water Resource Management 1, 315-332.
15. Hsieh, J.C.C. et al., 1998. Oxygen isotopic composition of soil water: quantifying evaporation and transpiration. Geoderma 82, 269-293.
16. Koch, P.L., 1998. Isotopic reconstruction of past continental environments. Annual Review of Earth and Planetary Sciences 26, 573-613.
17. Fox, D.L. et al., 2012. Pedogenic carbonate stable isotope record of environmental change during the Neogene in the southern Great Plains, southwest Kansas, USA: Oxygen isotopes and paleoclimate during the evolution of C4-dominated grasslands. GSA Bulletin 124, 431-443.
18. Birkeland, P.W., 1999. Soils and Geomorphology. Oxford University Press, New York.
19. Wynn, J.G., 2000. Paleosols, stable carbon isotopes and paleoenvironmental

interpretation of Kanapoi, Northern Kenya. Journal of Human Evolution 39, 411-432.
20. Bown, T.M., Krause, M.J., 1987. Integration of channel and floodplain suites, developmental sequence and lateral relations of alluvial paleosols. USGS Staff Published Research. 201.
21. van Breemen, N., Buurman, P., 2002. Soil Formation. Kluwer Academic, Dordrecht.
22. Srivastava, P., 2001. Paleoclimatic implications of pedogenic carbonates in Holocene soils of the Gangetic Plains, India. Palaeogeography, Palaeoclimatology, Palaeoecology 172, 207-222.
23. Quade, J. et al. 2013. The clumped isotope geothermometer in soil and paleosol carbonate. Geochemica et Cosmochemica Acta 105, 92.
24. Hough, B.G. et al., 2014. Calibration of the clumped isotope geothermometer in soil carbonate in Wyoming and Nebraska, USA: implications for paleoelevation and paleoclimate reconstruction. Earth and Planetary Science Letters 391, 110-120.
25. Breeker, D.E. et al., 2009. Seasonal bias in the formation and stable isotopic composition of pedogenic carbonate in modern soils from central New Mexico, USA. Geological Society of America Bulletin 121, 630-640.
26. Monger, H.C. et al., 2009. Scale and the isotopic record of C_4 plants in pedogenic carbonate: from the biome to the rhizosphere. Ecology 90, 1498-1511.
27. Wynn, J.G., 2004. Influence of Plio-Pleistocene aridification on human evolution: evidence from paleosols of the Turkana Basin, Kenya. American Journal of Physical Anthropology 123, 106-118.
28. Quinn, R.L. et al., 2007. Paleogeographic variations of pedogenic carbonate $\delta^{13}C$ values from Koobi Fora, Kenya: implications for floral compositions of Plio-Pleistocene hominin environments. Journal of Human Evolution 53, 560-573.
29. Quinn, R.L. et al., 2013. Pedogenic carbonate stable isotopic evidence for wooded habitat preference of early Pleistocene tool makers in the Turkana Basin. Journal of Human Evolution 65, 65-78.
30. Levin, N.E. et al., 2011. Paleosol carbonates from the Omo Group: isotopic records of local and regional environmental change in East Africa. Palaeogeography, Palaeoclimatology, Palaeoecology 307, 75-89.
31. Retallack, G.J., 2005. Pedogenic carbonate proxies for amount and seasonality of precipitation in paleosols. Geology 33, 333-336.
32. Driese, S.G., Mora, C.I., 1993. Physico-chemical environment of carbonate formation, Devonian vertic paleosols, central Appalachians. U.S.A. Sedimentology 40, 199-216.
33. Liutkus, C.M. et al., 2005. Paleoenvironmental interpretation of lake-margin deposits using $\delta^{13}C$ and $\delta^{18}O$ results from early Pleistocene carbonate rhizoliths, Olduvai Gorge, Tanzania. Geology 33, 377-380.
34. McDougall, I. et al., 2012. New single crystal $^{40}Ar/^{39}Ar$ ages improve time scale for deposition of the Omo Group, Omo-Turkana Basin, East Africa. Journal of Geological Society London 169, 213-226.
35. Brown, F.H., Feibel, C.S., 1991. Stratigraphy, depositional environments and palaeogeography of the Koobi Fora Formation. In: Harris, J.M. (Ed.), Koobi Fora

Research Project: Volume 3, Stratigraphy, Artiodactyls and Palaeoenvironments. Claredon Press, Oxford, pp. 1-30.
36. Hillhouse, J.W. et al. 1986. Magnetostratigraphy of the Koobi Fora Formation, Lake Turkana, Kenya. Journal of Geophysical Research 91, 11581-11595.
37. Gathogo, P.N., Brown, F.H., 2006. Stratigraphy of the Koobi Fora Formation (Pliocene and Pleistocene) in the Ileret region of northern Kenya. Journal of African Earth Sciences 45, 369-390.
38. Channell, J.E.T. et al., 2010. Reconciling astrochronological and 40Ar/39Ar ages for the Matuyama-Brunhes boundary and late Matuyama Chron. Geochemistry, Geophysics, Geosystems 11, Q0AA12.
40. Cerling, T.E. et al., 1988. An isotopic study of a fluviolacustrine sequence: the Plio-Pleistocene Koobi Fora sequence, East Africa. Palaeogeogr. Palaeoclimatol. Palaeoecol. 63, 335-356.
41. Patterson, D.B. et al., 2019. Comparative isotopic evidence from East Turkana supports a dietary shift within the genus Homo. Nat. Ecol. Evol.
42. White, F., 1983. The Vegetation of Africa, A Descriptive Memoir to Accompany the UNESCO/AETFAT/UNSO Vegetation Map of Africa. In: Natural Resource Research, vol. 20. UNESCO, pp. 1-356.
43. Levin, N.E., 2015. Environment and climate of early human evolution. Annual Review of Earth and Planetary Sciences 43, 405-429.
44. Faith, T. J. et al., 2018. Plio-Pleistocene decline of African megaherbivores: No evidence for ancient hominin impacts. Science, 362, 938–941.
45. Sikes, N.E. et al., 1999. Early Pleistocene habitat in Member 1 Olorgesailie based on paleosol stable isotopes. J. Hum. Evol. 37, 721 746.
46. Levin, N.E. et al., 2004. Isotopic evidence for Plio-Pleistocene environmental change at Gona Ethiopia. Earth and Planetary Science Letters 219, 93-110.
47. Quinn, R.L., Lepre, C.J., 2020. Revisiting the pedogenic carbonate isotopes and paleoenvironmental interpretation of Kanapoi. Journal of Human Evolution 140, 102549.
48. Li, M. et al., 2019. Acycle: Time-series analysis software for paleoclimate projects and education. Computing Geosciences 127, 12-22.
49. Ruggieri, E., 2013. A Bayesian approach to detecting change points in climatic records. International Journal of Climatology 3, 520-528.
50. Levin, N.E. et al., 2009. Isotopic composition of waters from Ethiopia and Kenya: insights into moisture sources for eastern Africa. Journal of Geophysical Research 114.
51. Feibel, C.S., 2011. A geological history of the Turkana Basin. Evolutionary Anthropology 20, 206-216.
52. Nutz, A. et al. 2020. Plio-Pleistocene sedimentation in West Turkana (Turkana Depression, Kenya, East African Rift System): paleolake fluctuations, paleolandscapes and controlling factors. Earth-Science Reviews 211, 103415.
53. Ludecke, T. et al., 2016. Persistent C3 vegetation accompanied Plio-Pleistocene hominin evolution in the Malawi Rift (Chiwondo Beds, Malawi). Journal of Human Evolution 90, 163-175.
54. Potts, R. et al., 2018. Environmental dynamics during the onset of the Middle Stone Age in eastern Africa. Science 360, 86-90.

55. Plummer, T.W. et al., 2009. Oldest evidence of tool-making hominins in a grassland-dominated ecosystem. Plos One 4, e7199.
56. Wood, B., Constantino, P. *Paranthropus boisei*: Fifty years of evidence and analysis. *Yrbk. Phys. Anthropol.* **50**, 106–132 (2007).
57. Dominguez-Rodrigo, M. et al., 2013. First partial skeleton of a 1.34-million-year-old *Paranthropus boisei* from Bed II, Oluvai Gorge, Tanzania. PLoS ONE 8, e80347.
58. Koch, C.F., Morgan, J.P., 1988. On the expected distribution of species' ranges. Paleobiology 14, 126–138.
59. Bobe, R., Leakey, M.G. 2009. Ecology of Plio-Pleistocene mammals in the Omo-Turkana Basin and the emergence of Homo. In F.E. Grine et al. (eds.), The First Humans: Origin and Early Evolution of the Genus Homo, 173 Vertebrate Paleobiology and Paleoanthropology, Springer
60. Wood, B.A., Patterson, B.A., 2020. *Paranthropus* through the looking glass. Proceedings of the National Academy of Sciences, USA 117, 23202-23204.
61. Quinn, R.L., 2015. Influence of Plio-Pleistocene basin hydrology on the Turkana hominin enamel carbonate $\delta^{18}O$ values. Journal of Human Evolution, 86, 13-31.
62. Frost, S.R. et al., 2017. New cranium of the large cercopithecid primate *Theropithecus oswaldi leakeyi* (Hopwood, 1934) from the paleoanthropological site of Makuyuni, Tanzania. Journal of Human Evolution 109, 46-56.
